# Spatial Organization and Coordination of the Plant Circadian System

**DOI:** 10.3390/genes12030442

**Published:** 2021-03-20

**Authors:** Maria A. Nohales

**Affiliations:** Instituto de Biología Molecular y Celular de Plantas (IBMCP), Consejo Superior de Investigaciones Científicas—Universidad Politécnica de Valencia, 46022 Valencia, Spain; manozaf@ibmcp.upv.es

**Keywords:** circadian clock, plant, tissue-specific, coupling, spatial organization, organismal synchronization

## Abstract

The plant circadian clock has a pervasive influence on many aspects of plant biology and is proposed to function as a developmental manager. To do so, the circadian oscillator needs to be able to integrate a multiplicity of environmental signals and coordinate an extensive and diverse repertoire of endogenous rhythms accordingly. Recent studies on tissue-specific characteristics and spatial structure of the plant circadian clock suggest that such plasticity may be achieved through the function of distinct oscillators, which sense the environment locally and are then coordinated across the plant through both intercellular coupling and long-distance communication. This review summarizes the current knowledge on tissue-specific features of the clock in plants and their spatial organization and synchronization at the organismal level.

## 1. Introduction

A circadian clock is an endogenous molecular mechanism that generates 24 h rhythms in a wide array of biological processes. As a consequence of the Earth’s rotation, organisms have evolved these timing mechanisms to align their physiology and development with the periodic changes in environmental conditions that occur over the day-night cycle. In the natural environment, the ability to track time enables organisms to anticipate these conditions and adequately coordinate different processes to occur at the most appropriate times. The anticipatory behavior conferred by these biological oscillators thus allows for an efficient distribution and use of metabolic resources and is thought to provide an adaptive advantage [1]. In fact, plant circadian mutants with dysfunctional clocks display reduced photosynthesis, growth and viability, especially under challenging conditions [1,2,3]. While the internal circadian oscillator runs with an intrinsic free-running period of approximately 24 h, it is synchronized, or entrained, to the exact period of environmental cycles through its sensitivity to multiple input signals, both exogenous and endogenous [4]. Light and temperature play a major role in the entrainment of the plant clock [5], which is also affected by other factors including humidity [6], ions [7,8] and metabolites [9,10].

**Figure 1:**
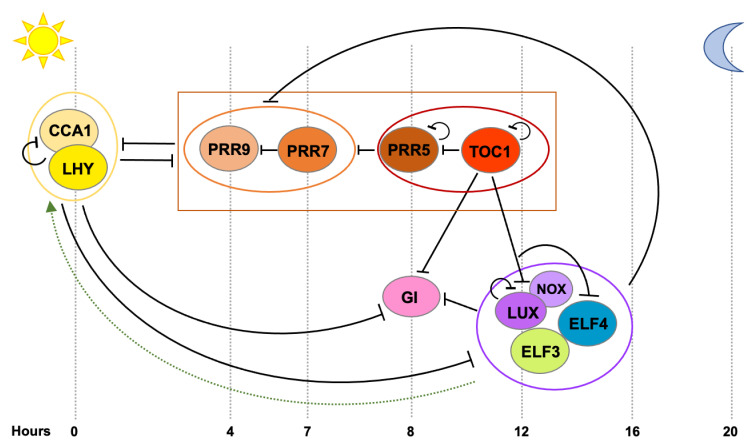
Transcriptional feedback loops at the core of the circadian oscillator in *Arabidopsis thaliana*. Clock components are sequentially expressed across the day as depicted from left to right. Black bars indicate repression of transcription and the broken green arrow, activation of transcription not proven to be direct. At dawn, CCA1 and LHY repress the expression of the *PRRs*, *GI* and the members of the Evening Complex (EC) *LUX*, *ELF3* and *ELF4*. PRR9, PRR7, PRR5 and TOC1 are sequentially expressed and repress the expression of *CCA1* and *LHY*, as well as each other’s. In the evening, TOC1 represses *CCA1*, *LHY* and the *PRRs*, as well as *GI*, *LUX* and *ELF4*. Later, the EC maintains repression on *GI*, *PRR9* and *PRR7* and likely indirectly activates *CCA1* and *LHY*.

Almost every aspect of plant physiology and development is subject to some extent of circadian regulation and many efforts have been devoted towards the identification of the genes and proteins that constitute the core molecular mechanism driving this pervasive rhythmicity. As a result, multiple clock components and intricate reciprocal regulatory connections have been identified [5,11,12] (Figure 1). Similarly to other organisms [13], the central oscillator in plants is composed of numerous transcriptional-translational loops where clock genes exert feedback regulation on each other, providing this timing mechanism and ultimately driving rhythmic expression of a significant portion of the transcriptome [14,15]. Two single MYB-domain transcription factors, CIRCADIAN CLOCK ASSOCIATED 1 (CCA1) and LATE ELONGATEDHYPOCOTYL (LHY), are expressed at dawn and they directly repress the expression of morning- and evening-phased clock genes, as well as their own expression [16,17]. As the day progresses, members of the PSEUDO-RESPONSE REGULATOR (PRR) family (PRR9, PRR7, PRR5 and PRR1 (better known as TIMING OF CAB EXPRESSION 1, TOC1)) are sequentially expressed and they repress *CCA1* and *LHY*, as well as each other [18,19,20]. In the evening, TOC1 represses all the previously expressed and aforementioned clock components, including *GIGANTEA* (*GI*), *LUX ARRYTHMO* (*LUX*) and *EARLY FLOWERING 4* (*ELF4*) [20]. Later during the night, a tripartite complex conformed by ELF3, ELF4 and LUX (the Evening Complex, EC) maintains the repression of *GI* and represses *PRR9*, *PRR7* and *LUX* and likely indirectly induces *CCA1* and *LHY* expression [21].

It is assumed that essentially every plant cell contains an autonomous circadian oscillator. However, different parts of the plant are in very different environments, perceive different environmental cues and are relevant to different biological processes. This heterogeneity translates in a variability in the individual cellular circadian rhythms, which need to be adequately integrated and coordinated both locally and across the plant to orchestrate organismal responses (see [22,23] for further review). Recent studies on plant circadian clock structure and intercellular relationships have not only put in context previous observations on circadian coupling and entrainment in plants, but they also provide an important spatial framework for future plant circadian studies. This review focuses on our current understanding of how the plant circadian clock is spatially organized and how separate tissue-specific clocks across the plant may communicate with each other and be synchronized at the organismal level. Future directions in plant circadian research are also discussed.

## 2. Tissue-Specificity of the Plant Circadian Clock

### 2.1. Early Evidences for Tissue-Specific Clocks

The existence of multiple oscillators in plants has long been proposed. Early studies in bean plants evidenced that the free-running period of stomatal opening and photosynthesis was different to that of leaflet movement [24] and studies in tobacco plants showed that rhythms in cytosolic free calcium (Ca^2+^) levels also free-run with a different period than the expression of a light-harvesting complex (*Lhc*)*b* gene family member [25]. A single clock can control multiple rhythms with different phases, but it will only render one period, as period is an intrinsic property of the oscillator. Hence, it was deduced that the difference in free-running periods displayed by these rhythms arose from the function of multiple separate plant oscillators with different intrinsic frequencies. Tissue-specific properties of these pacemakers were further investigated by analyzing a single rhythm, namely cytosolic free Ca^2+^ oscillations, in different tissues [26]. For this, transgenic tobacco plants expressing the aequorin protein (a luminescent reporter for Ca^2+^ levels) driven by different promoters with distinct spatial patterns of expression were generated. Under free-running conditions, circadian oscillations in Ca^2+^ exhibited distinct phases in each line. While these findings do not necessarily imply the function of separate oscillators, they evidence the existence of distinct cellular control mechanisms contributing to circadian rhythms in Ca^2+^ levels [26].

To inspect whether the different oscillators were composed of similar components or not, the effect of mutations in the central oscillator and light input pathway genes on presumably independent rhythms was analyzed. These rhythms comprised oscillations in cytosolic Ca^2+^ levels, as well as in *CHALCONE SYNTHASE* (*CHS*), *CHLOROPHYLL A/B BINDING PROTEIN* (*CAB*) and *PHYTOCHROME B* (*PHYB*) promoter activity, which owing to their distinct spatial distribution patterns and free-running periods were suggested to be regulated by separate oscillators located in different cell types [27,28,29]. Genetic analyses revealed that two clock-affecting mutations (in the core clock gene *TOC1* and the light signaling component *DE-ETIOLATED 1*, *DET1*) similarly affected the period of *CHS* and *CAB* promoter activity [27]. Likewise, misexpression of the red-light photoreceptor *PHYB* and the core clock genes *CCA1*, *LHY* and *ELF3* also affected the period of both *CAB* and *PHYB* promoter activity in a similar fashion, hence suggesting that the separate oscillators share common components [28]. Further supporting this notion, it was observed that rhythms in cytosolic Ca^2+^ levels and *CAB* promoter activity were both dependent on *CCA1*, *LHY* and *TOC1* function [29]. However, this study also revealed that a semidominant allele of *TOC1* (*toc1-1*), which contains an amino acid change in the conserved CCT (for CONSTANS, CONSTANS-LIKE and TOC1) domain, uncoupled both rhythms and only affected *CAB* oscillations [29]. Thus, these findings indicated that although the separate oscillators do seem to share common components, these may function or relate to each other differently in each tissue to render different frequencies.

### 2.2. Mechanisms Underlying Tissue-Specific Circadian Rhythms

Local differences in circadian rhythmicity from a similar oscillator can be achieved through various mechanisms including diverging levels in core clock gene expression, functional modulation of these clock genes by tissue-specific regulators and/or through differential perception of input signals.

The majority of clock genes are rhythmically expressed across the entire plant [30,31,32,33], but tissue-specific expression levels and circadian properties have been reported for many of them. Comparison of *CCA1* promoter activity under free-running conditions in the center of the leaf with that in the center of the rosette of *Arabidopsis thaliana* plants revealed differences in period length depending on the organ [34]. *CCA1* was also reported to display lower expression levels and a longer free-running period in guard cells compared to whole leaves [35]. A similar behavior was also observed for other oscillator components such as *LHY*, *TOC1* and *CCA1 HIKING EXPEDITION* (*CHE*) [35]. Interestingly, in the same study it was observed that another clock gene, *GI*, also had a later phase and run with a longer period in guard cells, but displayed similar expression levels in this cell type compared to whole leaves [35]. Hence, individual clock components behave differently across the plant. In terms of expression levels, *GI* seems to be more highly expressed in the vasculature [36], similarly to *PRR3*, which is suggested to regulate TOC1 protein stability in this tissue [31]. Further evidence on tissue-specific variations in the expression levels of core clock components was later provided by a genome-wide gene expression analysis in isolated vasculature and mesophyll cells compared to whole leaves [37]. It was observed that morning expressed clock genes such as *CCA1*, *LHY*, *PRR9* and *PRR7* are more highly expressed in mesophyll, while expression of evening phased genes such as *TOC1*, *ELF4* and *LUX* is higher in the vasculature [37]. Differences in the expression levels of clock genes have also been observed between shoots and roots [38]. Interestingly, morning and evening phased clock components seem to have a varying impact on circadian function in roots compared to shoots and mutation of several such components affects clock function differently in each organ [38,39,40,41,42], which indicates that the clock network might be wired differently in each case. Hence, divergence in the expression levels and tissue-specific molecular connections among core clock components is likely one of the mechanisms through which distinct local rhythms are achieved.

Differential processing of environmental signals is another factor that may contribute to tissue-specific circadian regulation. Because different parts of the plant are exposed to different microenvironments, it is anticipated that the impact of specific environmental cues, such as light quality and quantity, temperature or nutrient levels, will differ. Local differences in the clock’s sensitivity to a wide array of signals would enhance plasticity and could allow the clock to better adapt to ambient conditions locally [4,43].

Perhaps the most important entraining signal in plants is light. Plants use different classes of photoreceptors to sense the light environment and set the clock to the actual pace of day-night cycles [44]. These photoreceptors include PHYs and CRYPTOCHROMEs (CRYs), which transmit red and blue light signals, respectively [44]. Mutations in both PHY and CRY photoreceptors have been shown to affect circadian period length in response to different light qualities [45,46] and the spatial expression pattern of *PHY* and *CRY* genes varies among tissues [47,48,49]. Therefore, local differences in the sensitivity to light via these photoreceptors could be part of the mechanism underlying tissue-specific functions of the clock. Recent reports suggest that the differences in period length between shoots and roots can in fact be explained by different light inputs [38,50], in addition to other input signals such as metabolic sugars [50]. While the free-running period in roots and shoots is fairly similar under constant darkness, the period length of the root clock is considerably longer than the one in shoots under constant light conditions. Furthermore, the root clock is slowed down by blue light compared to red light, whereas the shoot clock showed similar periods in both blue and red light, evidencing differences in light perception and/or signal transduction in both organs [38]. Recent data suggest that function of the evening complex may in fact be part of the light input mechanism that differs between roots and shoots [41]. Additionally, tissue-specific functions of PHYB [51,52] and CRY2 [53], as well as other light signaling components that affect light input to the clock, such as COP1 [54], SPA1 [55] and PIFs [56,57,58], have been reported and could therefore contribute to local differences in the response to light. Further investigation will be required to uncover the overall topology of these tissue-specific light input networks and mechanistically define their function in clock rhythmicity.

In addition to light, temperature is another signal that conveys important information about the surrounding environment. Early studies showed that two separate oscillators involved in the regulation of *CAB2* and *CATALASE 3* (*CAT3*) expression had different sensitivity to light and temperature. The pacemaker regulating *CAB2* gene expression seemed to preferentially respond to light–dark cycles, while the one controlling *CAT3* expression, was more sensitive to temperature signals [59]. More recent studies have also reported differential processing of light and temperature signals in different tissues. By analyzing *TOC1* promoter activity oscillations in the vasculature compared to whole leaves under light-dark and temperature cycles, it was seen that the vascular clock has lower sensitivity to temperature and higher sensitivity to photoperiodic signals [60]. In fact, an oscillator located in vascular phloem companion cells plays an essential role in photoperiodic flowering control [37,61]. Conversely, a clock in the epidermis seems to display a higher sensitivity to ambient temperature and be required to coordinate other output processes such as temperature-dependent cell elongation [61]. Differences in the response to temperature between shoots and roots have also been documented. Temperature seems to have a more prominent effect on clock speed in roots and this is likely dependent on *PRR9* and *PRR7* function [39,40].

Altogether, several pieces of evidence suggest that local differences in circadian function may arise from a combination of factors, including heterogeneity in the expression levels of core clock components, tissue-specific connections within the circadian network and differential sensitivity and processing of environmental input signals.

## 3. Coordination of Tissue-Specific Clocks across the Plant

As outlined above, the overall circadian system in plants seems to be spatially organized with the clocks in different tissues displaying distinct circadian properties (such as period and phase) and responding differently to environmental cues. These observations necessarily raise the question of how these clocks communicate with each other and how they are coordinated to achieve optimal regulation of biological rhythms at the organismal level. Although initially proposed to be functionally independent [62], several studies have shown that the different cellular circadian oscillators across the plant indeed present a certain degree of local coupling, the strength of which varies depending on their location in the plant [34,35,37,50,63,64,65,66,67]. For example, coupling between clocks in different parts of the leaves seems to be rather weak [34,35,64,66], whereas cells within the shoot apex [65] and the root tip [63,67] are tightly coupled to each other and are able to maintain synchronization of oscillations longer under free-running conditions.

In terms of organization, analysis of the regulatory relationships between the clocks in two different tissues, mesophyll and vasculature, revealed the existence of a certain hierarchy among tissue-specific clocks. Perturbation of circadian rhythms in the vasculature (through overexpression of CCA1 in this tissue) affected the rhythmicity of both the vasculature and mesophyll clocks, while disruption of the mesophyll clock only affected rhythms in this cell-type [37]. These findings evidence that distinct local clocks are able to communicate with each other and that, under these conditions, the oscillator in the vasculature exerts a dominant function in the regulation of the adjacent mesophyll clock. Moreover, rhythms in the vasculature were observed to be notably robust compared to whole leaves [37]. Long-range asymmetric relationships between shoots and roots have also been reported. It was observed that circadian oscillations in roots can be entrained by signals derived from shoots and, hence, dominance of the shoot over the root clock was proposed early on [68]. More recently, investigations using ablation and micrografting experiments have reinforced the idea that a clock in the shoot, and more precisely in the shoot apex, drives rhythms across the plant. It was seen that disruption of the shoot clock using these techniques strongly affected rhythms in roots and, more importantly, that aberrant rhythms in mutant rootstocks could be partially recovered by grafting wildtype shoot apexes [65]. Of note is that the clock in the shoot apex was seen to present an outstanding level of synchrony compared to the clocks in other locations and this strong coordination seemed to be dependent on tight intercellular coupling at the shoot apex, which confers robustness against diverse perturbations [65]. Because these observations resemble characteristics of the circadian system in mammals, where a central clock in the suprachiasmatic nucleus drives rhythms in peripheral tissues [13], it was proposed that the plant clock is similarly hierarchically organized and that shoot apexes play a dominant role within this structure [65]. However, in addition to this strong synchronizing function of the shoot apex, the existence of further coordination centers within the plant circadian system has been suggested [22]. Such a decentralized structure would explain observations such as tissue-specific regulation of output processes independently of the shoot apex [37,61], as well as differences in the integration of environmental cues by distinct spatially separated oscillators as discussed earlier. Consistent with this view, newer investigations on the spatial structure of the plant clock at the organismal level [50,67] indeed indicate the presence of several coordination points across the plant. Using quantitative time-lapse microscopy, circadian rhythms in the accumulation of fluorescently tagged CCA1 protein were monitored across Arabidopsis seedlings at single cell resolution [67]. As in previous studies [65,68], differences in the robustness of the clock were observed among tissues, with the cotyledon and hypocotyl displaying greater synchronization and period similarity between cells than the root. Notably and similarly to the shoot apical clock, coupling between cells appeared to be remarkably stronger in the root tip. In terms of phase, CCA1 was observed to peak earlier in the upper portion of the hypocotyl compared to cotyledons and the lower hypocotyl, with the phase becoming further delayed down the root. However, a phase delay was also observed moving up from the root tip. Hence, there seem to be two waves of clock activity, one going down and one going up the root, which supports the existence of at least two coordination points across the plant.

Mathematical modelling together with experimental evidence suggest that intrinsic differences in period length together with cell-to-cell coupling may be sufficient to generate these waves in gene expression and coordinate the circadian system across the plant [50,67]. Hence, cell-to-cell communication may play an important role in the coordination of the different local rhythms, which, as outlined in the previous section, may arise from a number of reasons including differential sensitivity to input signals such as light [38,50], temperature [59,61] and metabolites [50]. Nevertheless, in addition to intercellular signaling, long-range communication may also contribute to clock coordination, as suggested by several studies. Disruption of circadian rhythms in roots by shoot apex ablation and micrografting [65] implies the existence of a long-distance synchronizing signal from the shoot, which has long been hypothesized to be photosynthesis related [68]. In addition, light piped down the root has been shown to affect entrainment in this organ and hence light piping from shoots to roots has been proposed as another mechanism that may contribute to their synchronization [69]. Finally, long-distance translocation of clock proteins has also been shown to mediate the shoot-to-root dialogue. As identified in a recent study [42], ELF4 protein moves from shoots to roots and affects circadian rhythmicity in this organ. Interestingly, movement of ELF4 is temperature-dependent and it is reduced at high temperatures, resulting in lower ELF4 accumulation and a faster clock in roots. Hence, ELF4 mobility represents a direct mechanism through which shoots would convey temperature information to the roots and affect clock function in this organ. The source of mobile ELF4, as well how it moves and whether it also affects local coupling, remains to be explored.

## 4. Concluding Remarks

**Figure 2:**
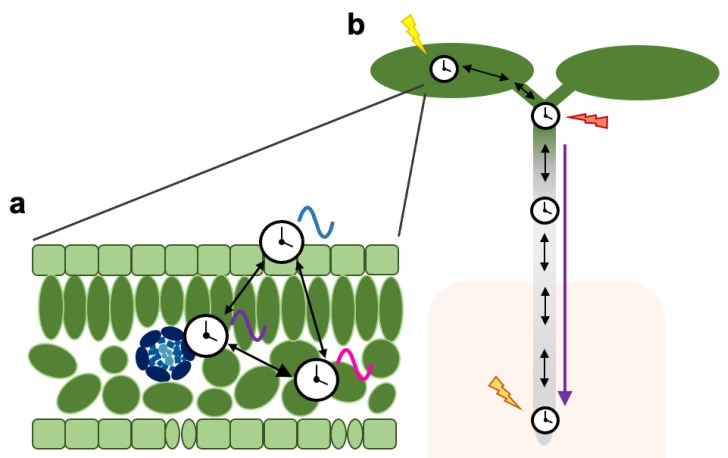
Schematic representation of spatial features of the plant circadian system. Black arrows indicate intercellular/inter-tissue communication. The purple arrow indicates long-distance communication. (**a**) Clocks in different tissues such as the vasculature, the mesophyll or the epidermis have distinct circadian characteristics and regulate different outputs (represented by colored waves), but are coordinated through local coupling mechanisms. In some instances, asymmetric relationships exist where the rhythmicity in one tissue strongly affects rhythms in neighboring cells. (**b**) Local clocks generate distinct rhythms partly due to their differential sensitivity to input signals (represented by colored lightning symbols). Heterogenous rhythms across the plant are coordinated through both intercellular coupling and long-distance communication.

It is becoming evident that the circadian system in plants is spatially structured and is composed by a complex web of distinct local clocks that communicate with each other and are coordinated across tissues and organs (Figure 2). As opposed to the centralized structure of the mammalian clock, the plant circadian system seems to be rather decentralized with multiple coordination points contributing to clock synchronization and entrainment. As sessile organisms, plants are continuously sensing their environment and different parts of the plant are exposed to very different conditions. Hence, such decentralization and regulatory complexity would allow for an increased flexibility towards input signals (sensed at different locations), while simultaneously maintaining the robustness of oscillations through intercellular coupling.

At least two points of coordination have been proposed, one in the shoot apex and one in the root tip [65,67]. While both seem to display similar circadian properties, the input signals to which they are entrained are yet to be defined, as well as whether additional clock hubs exist within the organismal clock network. Furthermore, because differential perception of input signals seems to be a major cause for circadian heterogeneity [38,50], it will be interesting to investigate the extent to which specific tissues contribute to overall circadian coordination in response to different signals. For example, the epidermis seems to be especially sensitive to temperature information, while the clock in the vasculature is required to integrate photoperiodic signals [37,60,61]. Along the same lines, mesophyll cells are the main sites of photosynthesis and may therefore represent a major source of photosynthetic entrainment [9,70]. Tissue-specific investigation of the mechanisms underlying this differential sensitivity to environmental signals (for example in light perception) will also be important.

Distinct circadian properties of local oscillators may additionally arise from differences in the wiring and function of clock components, for example through the existence of tissue-specific regulators. In fact, tissue-specific functions of the clock in the regulation of output pathways such as cell elongation and photoperiodic flowering have been reported [37,61] and tissue-specific *cis* elements in the promoters of clock regulated genes have been identified [37]. Current knowledge on core clock protein function and targets mostly arises from experiments performed on whole plants and hence it is likely that relevant tissue-specific characteristics have been overlooked. It will therefore be important to incorporate the spatial dimension in future studies on clock protein function, modulators and targets. Given the influence of circadian regulation on traits of agronomical value [71], these studies will not only be relevant to circadian research, but also bear the potential to provide valuable information that can be leveraged towards directed biotechnological crop improvement.

Considering the complexity of natural environments, it is likely that an array of signals and mechanisms converge for the regulation of rhythms across the plant. In addition to the signals mentioned in the previous section, several other signaling molecules may be involved in clock synchronization, including ions [72], metabolites [50,65,68], hormones [73] and other mobile proteins or mRNAs [74,75,76,77]. Identification of these messengers and their contribution to local and long-range coupling will be of great interest to circadian studies and will also advance our understanding of how complex molecular networks are organized and coordinated across multicellular organisms to render optimal physiological responses in the natural environment.
